# Supplementary material for: Demographic amplification is a predictor of invasiveness among plants
Source: Nat Commun. 2019 Dec 6;10:5602. doi: 10.1038/s41467-019-13556-w (PMC6897985; doi:10.1038/s41467-019-13556-w)
Supplement: Supplementary file 1 — Supplementary Information [file 41467_2019_13556_MOESM1_ESM.pdf]

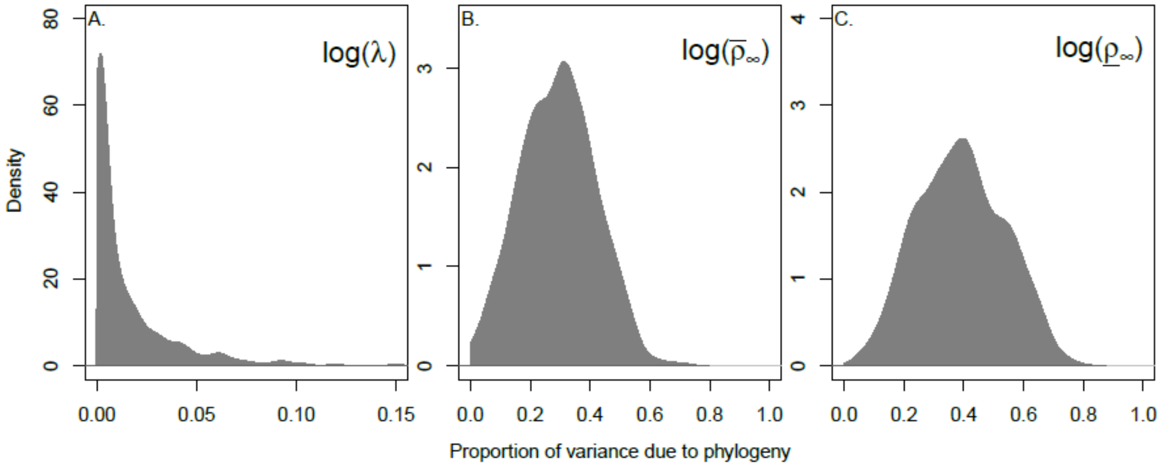

Suipplementary Figure 1: Phylogenetic signal in three demographic metrics. (A) stable rate of population increase (log (λ)); (B) upper bound on demographic inertia (log($\overline{\rho}_{\infty}$)) and (C) lower bound on demographic inertia (log($\underline{\rho}_{\infty}$)). Posterior, probability density distributions of the proportion of residual variance absorbed by the phylogenetic covariance matrix in the MCMCglmm models represented in Figure 2 of the main text. These figures demonstrate that phylogenetic signal in stable population growth rate is not credibly different from zero (A). However, we see credible non-zero phylogenetic signal in demographic amplification (B), and in demographic attenuation (C).

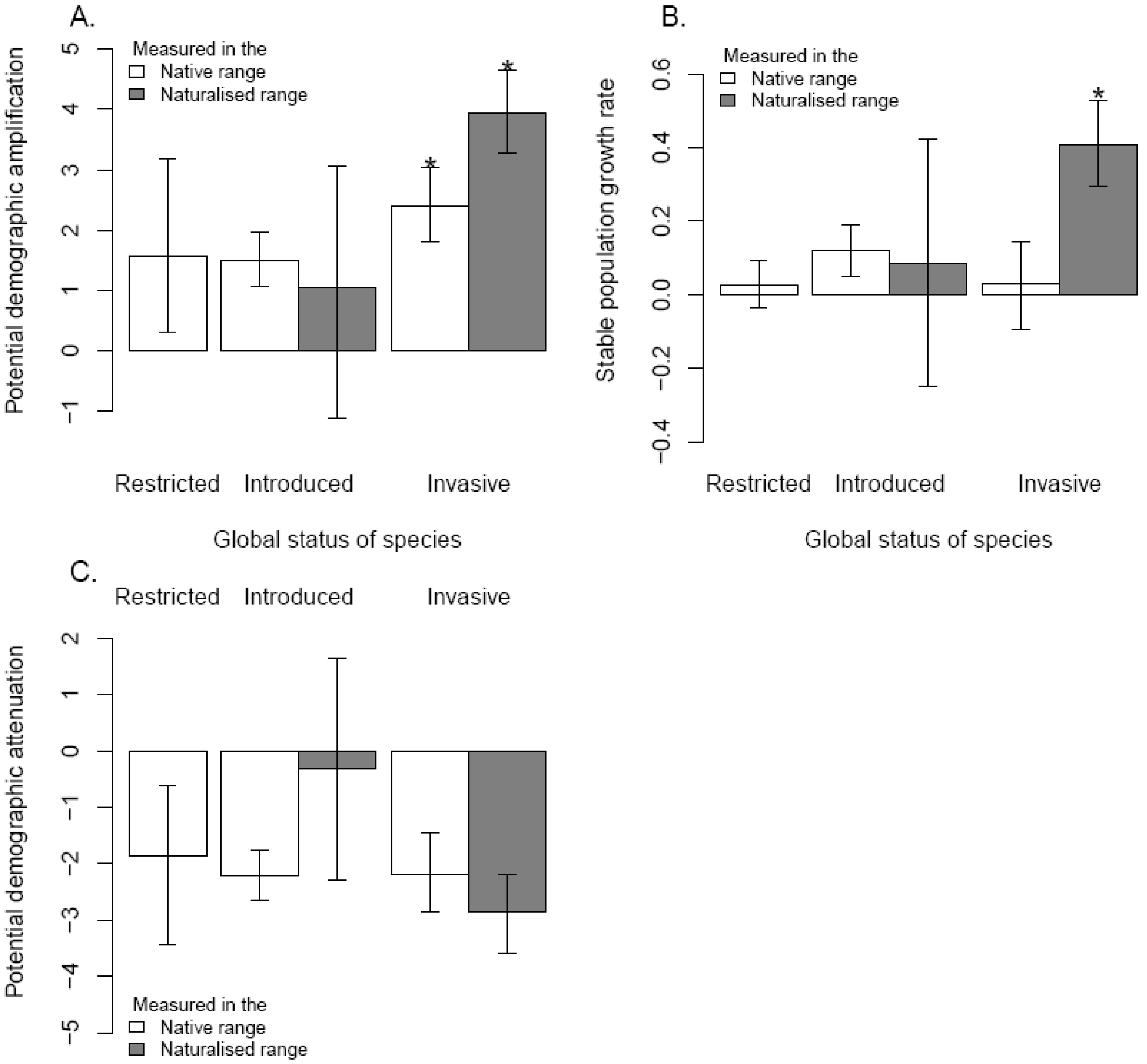

Supplementary Figure 2: Demographic traits of invasive and non-invasive plant populations. Bars show mean demographic trait (+/- 95% Credible Intervals) for populations of restricted, introduced and invasive plants, measured in the respective native or naturalised range. These analyses include all populations of all plant species as survey units in a hierarchical mixed effects model (c.f. the per-species analysis presented in the main text). Asterisks show invasive categories that are credibly different from all non-invasive categories; A) potential for demographic recovery following disturbance (upper bound on demographic inertia; mean log($\overline{\rho}_\infty$)); B) stable rate of population increase (mean log (λ)); and C) potential to attenuate in abundance following demographic disturbance (lower bound on demographic inertia; mean log($\underline{\rho}_\infty$)). Source data are provided as a Source Data file.

Supplementary Table 1: Associations between invasive category and reasons for study. Contingency tables count the plant populations appearing as entries in COMPADRE where the source material is available, and categorise them into invasive categories and the reasons for study as cited by the original authors. There exists a bias towards the study of restricted species for their conservation value, although the majority of restricted species were studied for other reasons; and a bias towards the study of invasive species for their invasiveness or weediness, however several invasive species were studied for their basic interest value.

		Studied because invasive/weed		Studied because endangered	
Global Status	Where studied	No	Yes	No	Yes
Restricted	Native	193	2	125	70
Introduced	Native	82	2	68	16
Introduced	Naturalised	0	3	3	0
Invasive	Native	24	1	24	1
Invasive	Naturalised	11	12	22	1

Supplementary Table 2: Comparison of demographic metrics between categories of reason for study. Results show outcome of simple generalised linear model for each demographic metric, including *F*-ratio, degrees of freedom and *P*-value. None of these comparisons yielded significant differences, suggesting that the main manuscript's results describe real differences among categories, rather than biases caused by choice of study species.

Demographic Metric	Comparison of restricted species studied for conservation value vs not	Comparison of invasive species studied for invasiveness/ weediness vs not
Log(stable rate of increase)	$F_{1,46} = 1.362$, $P = 0.249$	$F_{1,46} = 0.003$, $P = 0.958$
Log(potential for demographic amplification)	$F_{1,46} = 0.098$, $P = 0.756$	$F_{1,46} = 0.031$, $P = 0.859$
Log(potential for demographic attenuation)	$F_{1,46} = 0.101$, $P = 0.753$	$F_{1,46} = 0.173$, $P = 0.678$
