# Supplementary material for: Demographic amplification is a predictor of invasiveness among plants
Source: Nat Commun. 2019 Dec 6;10:5602. doi: 10.1038/s41467-019-13556-w (PMC6897985; doi:10.1038/s41467-019-13556-w)
Supplement: Supplementary file 3 — Reporting Summary [file 41467_2019_13556_MOESM3_ESM.pdf]

nature research

Corresponding author(s): *Double-blind peer review submissions: write DBPR and your manuscript number here instead of author names.*

Last updated by author(s): *YYYY-MM-DD*

Reporting Summary

Nature Research wishes to improve the reproducibility of the work that we publish. This form provides structure for consistency and transparency in reporting. For further information on Nature Research policies, see Authors & Referees and the Editorial Policy Checklist.

Statistics

For all statistical analyses, confirm that the following items are present in the figure legend, table legend, main text, or Methods section.

n/a	Confirmed	
☐	☒	The exact sample size (n) for each experimental group/condition, given as a discrete number and unit of measurement
☐	☒	A statement on whether measurements were taken from distinct samples or whether the same sample was measured repeatedly
☐	☒	The statistical test(s) used AND whether they are one- or two-sided<br>*Only common tests should be described solely by name; describe more complex techniques in the Methods section.*
☐	☒	A description of all covariates tested
☐	☒	A description of any assumptions or corrections, such as tests of normality and adjustment for multiple comparisons
☐	☒	A full description of the statistical parameters including central tendency (e.g. means) or other basic estimates (e.g. regression coefficient) AND variation (e.g. standard deviation) or associated estimates of uncertainty (e.g. confidence intervals)
☐	☒	For null hypothesis testing, the test statistic (e.g. F, t, r) with confidence intervals, effect sizes, degrees of freedom and P value noted<br>*Give P values as exact values whenever suitable.*
☐	☒	For Bayesian analysis, information on the choice of priors and Markov chain Monte Carlo settings
☐	☒	For hierarchical and complex designs, identification of the appropriate level for tests and full reporting of outcomes
☐	☒	Estimates of effect sizes (e.g. Cohen's d, Pearson's r), indicating how they were calculated

Our web collection on statistics for biologists contains articles on many of the points above.

Software and code

Policy information about availability of computer code

Data collection	Software R version 3.5.1. All code provided as R Markdown document.
Data analysis	Software R version 3.5.1. All code provided as R Markdown document.

For manuscripts utilizing custom algorithms or software that are central to the research but not yet described in published literature, software must be made available to editors/reviewers. We strongly encourage code deposition in a community repository (e.g. GitHub). See the Nature Research guidelines for submitting code & software for further information.

Data

Policy information about availability of data

All manuscripts must include a data availability statement. This statement should provide the following information, where applicable:

- Accession codes, unique identifiers, or web links for publicly available datasets
- A list of figures that have associated raw data
- A description of any restrictions on data availability

Data Availability All data used for analyses are provided in online supplementary materials, alongside code for analysis. The COMPADRE database12 is published online https://www.compadre-db.org/. All data are available from authors on request.

Field-specific reporting

Please select the one below that is the best fit for your research. If you are not sure, read the appropriate sections before making your selection.

☐ Life sciences ☐ Behavioural & social sciences ☒ Ecological, evolutionary & environmental sciences

Ecological, evolutionary & environmental sciences study design

All studies must disclose on these points even when the disclosure is negative.

Study description	Comparative analysis of plant population projection matrices sourced from the COMPADRE database, categorised by region of study (native or naturalised) and global invasive status (restricted, naturalised, invasive). Sample sizes provided in manuscript. Phylogenetic regression performed on demographic metrics against study status and invasive status, with phylogenetic covariance sdtructure.
Research sample	Plant population demographic schedules from COMPADRE, filtered to include only unmanipulated populations.
Sampling strategy	All available populations in COMPADRE, which matched tips of the Plantae phylogeny, were used in analysis.
Data collection	Data collected by members of the COMPADRE database team, and verified by the COMPADRE committee, over several years.
Timing and spatial scale	No limits on timescale of data collection (data sourced from primary literature).
Data exclusions	Plant populations ecluded if species not included in Plantae phylogeny.
Reproducibility	All data published in primary or secondary literature, usually peer reviewed. Reproducibility confirmed using tests of robustness of analyses (see methods); also all scripts for analysis are provided for reproducibility.
Randomization	All available data were used. Risk of bias is discussed in manuscript.
Blinding	Blinding not possible since data gathered from authored publications.

Did the study involve field work? ☐ Yes ☒ No

Reporting for specific materials, systems and methods

We require information from authors about some types of materials, experimental systems and methods used in many studies. Here, indicate whether each material, system or method listed is relevant to your study. If you are not sure if a list item applies to your research, read the appropriate section before selecting a response.

Materials & experimental systems

n/a	Involved in the study
☒	☐ Antibodies
☒	☐ Eukaryotic cell lines
☒	☐ Palaeontology
☒	☐ Animals and other organisms
☒	☐ Human research participants
☒	☐ Clinical data

Methods

n/a	Involved in the study
☒	☐ ChIP-seq
☒	☐ Flow cytometry
☒	☐ MRI-based neuroimaging
