# Supplementary material for: Demographic amplification is a predictor of invasiveness among plants
Source: Nat Commun. 2019 Dec 6;10:5602. doi: 10.1038/s41467-019-13556-w (PMC6897985; doi:10.1038/s41467-019-13556-w)
Supplement: Supplementary file 4 — Description of Additional Supplementary Files [file 41467_2019_13556_MOESM4_ESM.pdf]

Description of Additional Supplementary Files

File Name: Supplementary Data 1
Description: Plant species used in analyses of links between demographic traits and invasiveness. Data table lists species' scientific names, original source of publication of the demographic models, the status (native or non-native) of the populations studied, the global status of the species (not introduced outside native range; introduced but not considered invasive; invasive in some part of the naturalized range), and the database used to identify global status.

File Name: Supplementary Data 2
Description: Demographic information used to analyse links between demographic traits and invasiveness. Data were extracted from COMPADRE database version 3.0.0, and filtered to include only unmanipulated populations. Associated metadata columns are described in detail at https://www.compadre-db.org/Home/Index.

File Name: Supplementary Software 1
Description: R Markdown script, executable script for R, and plant phylogeny .tre file used to replicate all data handling and mcmcGLMM analyses performed in this manuscript.
