# Supplementary material for: Demographic amplification is a predictor of invasiveness among plants
Source: Nat Commun. 2019 Dec 6;10:5602. doi: 10.1038/s41467-019-13556-w (PMC6897985; doi:10.1038/s41467-019-13556-w)
Supplement: Supplementary file 7 — Supplementary Software 1 [file 41467_2019_13556_MOESM7_ESM.zip › Jelbert et al Supplementary Software Markdown.pdf]

Supplementary Materials: Demographic predictors of invasiveness among plants

Kim Jelbert, Danielle Buss, Jenni McDonald, Stuart Townley, Miguel Franco, Iain Stott, Owen Jones, Roberto Salguero-Gómez, Yvonne Buckley, Tiffany Knight, Matthew Silk, Francesca Sargent, Simon Rolph, Phil Wilson & Dave Hodgson

04 June 2018

Contents

1. Supplementary methodological notes
2. R code to replicate the analyses in the paper
3. Guide to supplementary data files

1. Supplementary methodological notes

Robustness of Statistical Analyses

We chose to describe our analyses of demographic metrics (stable population growth, demographic inertia) using Monte Carlo Markov Chain generalised linear mixed models, because of the special features of our dataset. In our raw data, we have demographic indices for each population of each species in each year of measurement. These indices measure population dynamics, which project forward in time. To prevent conflation of time-dependent data and time-dependent metrics, we summarised each population's demography using a time-averaged population projection matrix (PPM). This yielded replicate population-level PPMs for each species. We needed a statistical modelling tool which can tease apart the phylogenetic non-independence of the data (species-level shared evolutionary history); repeated measures per species (multiple populations) and the fact that each species could contribute populations to more than one "population status" category (for example *Carduus nutans*, an invasive species, has been measured in both its native and its naturalised ranges). MCMCglmm is the best statistical algorithm, to our knowledge, that achieves valid analysis of this hierarchical design.

However, we recognise that a full analysis of demographic metrics measured at the population scale, with multiple populations per species, which themselves are patterned phylogenetically, is a weighty analysis that can be hard to explain in a restricted word count and for a wide readership. Also, an analysis that uses population as its experimental unit means that predictions must be made for populations, not for species. Hence we chose to report a simplified analysis that derived mean demographic metrics per species. This approach still required MCMCglmm because a small number of species were represented across multiple "population status" categories. The results of this "per species per status" analysis are presented in the manuscript. To check the robustness of the results, we performed the full-scale "per population per species" analysis and found patterns that matched the results presented (models presented in Extended Data). We also down-scaled the analyses presented here, first by removing species that were represented in multiple "population status" categories. This allowed us to use a generalised least squares regression model with phylogenetic correlation structure (PGLS) because each species was represented just once in the simplified dataset. The significance of the differences between categories, for each demographic metric, were the same as presented in the manuscript (presented in Extended Data). Throughout all the checks of alternative models, the same pattern of results emerged: stable population growth rates were highest (and significantly so) among species measured in the naturalised range, irrespective of their invasiveness. Meanwhile demographic inertia, describing the ability of populations to recover following disturbance, was highest (and significantly so) for invasive species, even when measured in their native range.

Finally, we have noted in other analyses of stable and transient population dynamics based on PPMs, that transient metrics (e.g. demographic inertia) can be influenced by the number of lifestages that are used to model structured life cycles. We re-analysed our models with the inclusion of a second predictor: dimension of the PPM itself. Matrix dimension did not influence the magnitude of stable rate of population growth, and its inclusion in the regression models did not affect the significance of the differences between invasiveness categories. Matrix dimension was a significant predictor of demographic inertia (the magnitude of the upper bound on inertia increased with increasing matrix dimension) but the inclusion of this significant predictor did not affect the significant differences among invasiveness categories Results of these extended regression models are presented in Extended Data.

2. R code to replicate the analyses in the paper

2.1 Set up

Load required R packages

```
library(popdemo)
library(popbio)
library(MCMCglmm)
library(ape)
library(nlme)
```

Read in data

- the adapted COMPADRE dataset
- the COMPADRE phylogeny

(*after setting the directory/path*)

```
direc<-"C:/Users/mjs245/Dropbox/Hodgson Postdoc (non-badger)/Plant Invasiveness paper/Rmarkdownfinal/"
```

```
compadre_full<-read.csv(paste0(direc,"COMPADRE filtered Jelbert et al 20180614.csv"), header=T)
tree<-read.tree(paste0(direc,"phylogeny_test.tre"))
```

Define required functions

Function to convert post-reproductive census projection matrix models to pre-reproductive census models

In post-reproductive models, the recruitment row describes a "seed" or "propagule" stage that is contributed into by reproductive adults which have survived for the preceding projection interval. To convert to pre-reproductive census, which is the more prevalent representation, recruitment rates are divided by their respective adult rates of survival and growth; then multiplied by rates of germination, survival and growth of propagules, and added to the relevant rows and columns of the converted projection matrix.

```
convert2pre <- function(mat,matF,matC,problem)
{
  if(problem=="pre"){mat_corrected_pre<-mat}else{
    mat <- Matlab2R(mat)
    matF <- Matlab2R(matF)
    matC <- Matlab2R(matC)
    matS<-mat-matF-matC
    matrix_size <- nrow(mat)
    if(problem=="error"){
      newmat<-matS%*%matF+matC+matS
    }
    if (problem=="post"){ # persistent seedbank
      surv_vec<-apply(matS,2,sum)
      surv_mat<-matrix(surv_vec,nrow=matrix_size,ncol=matrix_size,byrow=T)
      newmat1<-matF/surv_mat
      newmat<-matS%*%newmat1+matC+matS
    }
    if(mat[1,1]==0){
      mat_corrected_pre<-newmat[2:matrix_size,2:matrix_size]}else{
        mat_corrected_pre<-newmat}

    mat_corrected_pre<- R2Matlab(mat_corrected_pre)
  }
  return(mat_corrected_pre)
}
```

2.2 R code for conversion of projection matrix models to standardise analyse

Code uses the full PPM (mat), the fecundity part of the PPM (matF), and the clonal reproduction part of the PPM (matC), extracted from the COMPADRE database. Code consists of functions that (a) deal with the "seeds error", in which fecundity is falsely recorded as a full-year life cycle transition; and (b) converts post-reproductive census projection matrix models to pre-reproductive census models. (a) This function multiplies seed production by rates of seed survival, germination and growth, to better represent recruitment as the full-year life cycle transition. The outcome tends to reduce matrix dimension by a single life stage (removing the false "seed" stage). (b) In post-reproductive models, the recruitment row describes a "seed" or "propagule" stage that is contributed into by reproductive adults which have survived for the preceding projection interval. To convert to pre-reproductive census, which is the more prevalent representation, recruitment rates are divided by their respective adult rates of survival and growth; then multiplied by rates of germination, survival and growth of propagules, and added to the relevant rows and columns of the converted projection matrix.

Convert to pre-reproductive matrices from post-reproductive matrices and from those with error in the propagule stage

```
PPM<-as.character(compadre_full$matrix_a_string)
PPMfix<-PPM
F<-as.character(compadre_full$matrix_f_string)
C<-as.character(compadre_full$matrix_c_string)
pp<-compadre_full$fixed_census_timing
for(i in 1:dim(compadre_full)[1]){
  if(compadre_full$fixed_census_timing[i]%in%c("error","post")){PPMfix[i]<-try(convert2pre(PPM[i],F[i],C[i],
pp[i]))}
}
compadre_full$Afix<-PPMfix
compadre<-compadre_full
```

Subset rows of dataframe to remove seasonal and laboratory matrices

```
compadre<-compadre[(!compadre$matrix_composition=="NDY - Seasonal" & !compadre$population_ecoregion=="LAB"),
]
compadre<-compadre[(!compadre$matrix_treatment_type.1=="LAB"),]
```

Remove any rows with population_name that includes a semicolon

This means that:

- only keep individual matrices for populations with multiple matrices
- keep the mean matrix for those populations with only one matrix

```
compadre<-compadre[-grep(";",compadre$population_name,value=FALSE),]
```

(optional) Retain only unmanipulated treatment types

```
compadre<-compadre[compadre$matrix_treatment_type=="Unmanipulated",]
```

Create list of mean matrices for each population

(*2965 in total*)

```
PPM<-as.character(compadre$Afix)
PPMs<-as.list(numeric(length(PPM)))
Dimensions<-numeric(length(PPM))
Sp<-as.character(compadre$species_accepted)
Pop<-as.character(compadre$population_name)
for(i in 1:length(PPMs)){
  PPMs[[i]]<-Matlab2R(PPM[i])
  Dimensions[i]<-dim(PPMs[[i]])[1]
}
```

2.3 R code for the handling of the filtered COMPADRE dataset containing population projection matrices

Including information on taxonomy, invasive status, population, year and other ancillary information. Seasonal projection matrices and any "populations" measured in a laboratory setting have previously been filtered out. Populations experiencing experimental treatments have also already been filtered out. Aggregate PPMs, formed from individual population replicates, have also been pre-excluded. This section of code creates a mean matrix through time for each population and then filters out any reducible, imprimitive and non-ergodic matrices. The outcome is a dataset, with one row per population, that includes the mean matrix (averaged through time), and all associated taxonomic and status metadata.

The subsequent section of code aggregates dataframe information on species, matrix dimensions and location to add to the dataframe

First combine species with same dimensions and in same population location to create a unique id for each array

1. paste population site following species ID

2. paste dimension to ensure similar dimensions are compared across similar species

3. group to unique levels

```
Sp3<-paste(Sp,Pop,sep=".")
Sp3<-paste(Sp3,as.character(Dimensions),sep=".x")
Sp3<-as.factor(noquote(Sp3))
ID<-levels(Sp3)
```

Then create a list of arrays of replicated matrices:

- First create empty lists/vectors

```
aggrPPMs<-as.list(numeric(length(ID)))
reps<-numeric(length(ID))
orders<-numeric(length(ID))
for(i in 1:length(ID)){
  orders[i]<-mean(Dimensions[Sp3==ID[i]])
  reps[i]<-length(which(Sp3==ID[i]))
  aggrPPMs[[i]]<-numeric(orders[i]^2*reps[i])
  dim(aggrPPMs[[i]])<-c(orders[i],orders[i],reps[i])
}
```

- Then add aggregate matrices across duplicated matrices for each population for each species

```
for(i in 1:length(ID) ){
  for(j in 1:reps[i]){
    aggrPPMs[[i]][,,j]<-PPMs[Sp3==ID[i]][[j]]
  }}
```

Now merge meta-data with new aggregated matrices

```
spa<-paste(unique(Sp))
Species<-factor(length(ID),levels=c(spa))

ss<-paste(unique(compadre$population_invasive_status_study))
Status<-factor(length(ID),levels=c(ss))

se<-paste(unique(compadre$population_invasive_status_elsewhere))
Status.Else<-factor(length(ID),levels=c(se))

po<-paste(unique(compadre$taxonomy_order))
plant.order<-factor(length(ID),levels=c(po))

Year<-numeric(length(ID))
```

Create new empty fields/columns ready to store information in a reduced dataframe

Then fill these columns from the original dataframe

1. matrix treatment type

```
mtrt<-paste(unique(compadre$matrix_treatment_type))
matrix_treatment<-factor(length(ID),levels=c(mtrt))



for(i in 1:length(ID)){
  Species[i]<-(Sp[Sp3==ID[i]][1])
  Year[i]<-compadre$publication_year[Sp3==ID[i]][1]
  Status[i]<-compadre$population_invasive_status_study[Sp3==ID[i]][1]
  Status.Else[i]<-compadre$population_invasive_status_elsewhere[Sp3==ID[i]][1]
  plant.order[i]<-compadre$taxonomy_order[Sp3==ID[i]][1]
  matrix_treatment[i]<-compadre$matrix_treatment_type[Sp3==ID[i]][1]
}
```

This section of code removes some incorrect matrices from the analysis (NA/non-ergodic/non-irreducible/non-primitive)

```
mean.A<-vector("list", length(ID))
check.A<-numeric(length(ID))
for(i in 1:length(ID)){
  mean.A[[i]]<-apply(aggrPPMs[[i]],c(1,2),mean,na.rm=T)
  check.A[i]<-sum(mean.A[[i]])
}
mean.A<-mean.A[!is.na(check.A)]
ID<-ID[!is.na(check.A)]
check.matrices<-mean.A

erg<-factor(length(check.matrices),levels=c("TRUE","FALSE"))
irreducible<-factor(length(check.matrices),levels=c("TRUE","FALSE"))
primitivity<-factor(length(check.matrices),levels=c("TRUE","FALSE"))

for (i in 1:length(mean.A)){
  erg[i]<-is.matrix_ergodic(mean.A[[i]])
  irreducible[i]<-is.matrix_irreducible(mean.A[[i]])
  primitivity[i]<-is.matrix_primitive(mean.A[[i]])
}

delete<-which(erg=="FALSE")
delete1<-which(irreducible=="FALSE")
delete2<-which(primitivity=="FALSE")
del<-unique(c(delete,delete1,delete2))

mean.A<-mean.A[-del]
ID.check<-ID[-del]
A.string<-character(length(ID.check))
for(i in 1:length(ID.check)){
  A.string[i]<-R2Matlab(mean.A[[i]])}
```

We can now create multi_pop dataframe using the new variables and information from which matrices passed checks in the previous section of code

We also generate empty vectors to store the demographic response variables and then populate them

```
multi_pop<-data.frame(Species,Status,Status.Else,Year,plant.order,matrix_treatment)
multi_pop<-multi_pop[!is.na(check.A),]
multi_pop<-multi_pop[-del,]
multi_pop$ID.check<-ID.check

multi_pop$lambda.mean<-numeric(dim(multi_pop)[1])
multi_pop$inertiaup.mean<-numeric(dim(multi_pop)[1])
multi_pop$inertiadown.mean<-numeric(dim(multi_pop)[1])
multi_pop$reactivity.mean<-numeric(dim(multi_pop)[1])
multi_pop$maxatt.mean<-numeric(dim(multi_pop)[1])
multi_pop$maxamp.mean<-numeric(dim(multi_pop)[1])

for(i in 1:dim(multi_pop)[1]){
  multi_pop$lambda.mean[i]<-abs(eigen(mean.A[[i]])$values[1])
  multi_pop$inertiaup.mean[i]<-inertia(mean.A[[i]],bound="upper")
  multi_pop$inertiadown.mean[i]<-inertia(mean.A[[i]],bound="lower")
  multi_pop$reactivity.mean[i]<-reactivity(mean.A[[i]])
  multi_pop$maxatt.mean[i]<-maxatt(mean.A[[i]])
  multi_pop$maxamp.mean[i]<-maxamp(mean.A[[i]])
}
multi_pop$A<-A.string
```

2.4 R code to add phylogenetic data and prepare final dataframes for analysis

Data handling code to match phylogeny to per-species demographic indices, eventually producing the results offered in the main text. This code matches the species names in the dataset containing mean demographic indices per population with species names in the phylogeny. Mismatches are dealt with case-by-case, usually by removing subspecies status from the COMPADRE dataset. The code then calculates mean demographic indices (stable population growth, maximum amplification and minimum attenuation) for each species. Code calculates species' invasive status (restricted vs naturalised non-invasive vs invasive) and population status (native range vs naturalised range). This is not a factorial analysis because there are no representative species in the "restricted, measured in the naturalised range category" (such

populations cannot exist)

Rename any subspecies in the multi_pop dataset so that they can be found in the phylogeny

```
multi_pop$species_tree<-gsub(" ", "_", multi_pop$Species)

multi_pop$species_tree<-gsub("Adenocarpus_aureus_gibbsianus", "Adenocarpus_aureus", multi_pop$species_tree)
multi_pop$species_tree<-gsub("Adenocarpus_gibbsianus", "Adenocarpus_aureus", multi_pop$species_tree)
multi_pop$species_tree<-gsub("Anthyllis_vulneraria_alpicola", "Anthyllis_vulneraria", multi_pop$species_tree
)
multi_pop$species_tree<-gsub("Anthyllis_vulneraria_subsp_alpicola", "Anthyllis_vulneraria", multi_pop$specie
s_tree)
multi_pop$species_tree<-gsub("Antirrhinum_molle_lopesianum", "Antirrhinum_molle", multi_pop$species_tree)
multi_pop$species_tree<-gsub("Arenaria_grandiflora_bolosii", "Arenaria_grandiflora", multi_pop$species_tree)
multi_pop$species_tree<-gsub("Chamaecrista_lineata_keyensis", "Chamaecrista_lineata", multi_pop$species_tree
)
multi_pop$species_tree<-gsub("Betula_pubescens_pumila", "Betula_pubescens", multi_pop$species_tree)
multi_pop$species_tree<-gsub("Dodonaea_viscosa_angustifolia", "Dodonaea_viscosa", multi_pop$species_tree)
multi_pop$species_tree<-gsub("Echinospartum_ibericum_algibicum", "Echinospartum_ibericum", multi_pop$species
_tree)
multi_pop$species_tree<-gsub("Eriogonum_longifolium_gnaphalifolium", "Eriogonum_longifolium", multi_pop$spec
ies_tree)
multi_pop$species_tree<-gsub("Chamaecrista_lineata_keyensis", "Chamaecrista_lineata", multi_pop$species_tree
)
multi_pop$species_tree<-gsub("Gaura_neomexicana_coloradensis", "Gaura_neomexicana", multi_pop$species_tree)
multi_pop$species_tree<-gsub("Geonoma_pohliana_weddelliana", "Geonoma_pohliana", multi_pop$species_tree)
multi_pop$species_tree<-gsub("Gilia_tenuiflora_hoffmannii", "Gilia_tenuiflora", multi_pop$species_tree)
multi_pop$species_tree<-gsub("Leontopodium_nivale_alpinum", "Leontopodium_nivale", multi_pop$species_tree)
multi_pop$species_tree<-gsub("Lespedeza_juncea_sericea", "Lespedeza_juncea", multi_pop$species_tree)
multi_pop$species_tree<-gsub("Magnolia_macrophylla_dealbata", "Magnolia_macrophylla", multi_pop$species_tree
)
multi_pop$species_tree<-gsub("Pityopsis_aspera_aspera", "Pityopsis_aspera", multi_pop$species_tree)
multi_pop$species_tree<-gsub("Quercus_mongolica_crispula", "Quercus_mongolica", multi_pop$species_tree)
multi_pop$species_tree<-gsub("Silene_douglasii_oraria", "Silene_douglasii", multi_pop$species_tree)
multi_pop$species_tree<-gsub("Silene_glaucifolia_pseudoviscosa", "Silene_glaucifolia", multi_pop$species_tre
e)
multi_pop$species_tree<-gsub("Tragopogon_pratensis_subsp._Orientalis", "Tragopogon_pratensis", multi_pop$spe
cies_tree)
multi_pop$species_tree<-gsub("Tragopogon_pratensis_subsp._Pratensis", "Tragopogon_pratensis", multi_pop$spec
ies_tree)
multi_pop$species_tree<-gsub("Vella_pseudocytisus_paui", "Vella_pseudocytisus", multi_pop$species_tree)
multi_pop$species_tree<-gsub("Verticosa_staminosa_staminosa", "Verticosa_staminosa", multi_pop$species_tree)
multi_pop$species_tree<-gsub("Viola_sagittata_ovata", "Viola_sagittata", multi_pop$species_tree)
multi_pop$species_tree<-gsub("Alnus_incana_rugosa", "Alnus_incana", multi_pop$species_tree)
multi_pop$species_tree<-gsub("Escobaria_robbinsiorum", "Escobaria_robbinsorum", multi_pop$species_tree)
multi_pop$species_tree<-gsub("Mammillaria_napia", "Mammillaria_napina", multi_pop$species_tree)
multi_pop$species_tree<-gsub("Styrax_obassia", "Styrax_obassis", multi_pop$species_tree)
multi_pop$species_tree<-gsub("Verticosa_staminosa", "Verticordia_staminosa", multi_pop$species_tree)
```

Check phylogeny for species and create missing vector to store information on which species lack phylogenetic data (*now subspecies have been renamed*)

```
species_check<-unique(multi_pop$Species)
multi_pop$species_tree<-gsub(" ", "_", multi_pop$Species)
species_tree<-unique(tree$tip.label)
species_data<-unique(multi_pop$species_tree)
missing<-setdiff(species_data,species_tree)
```

Remove any rows of the multi_pop dataframe that don't have phylogenetic information

Create animal column/field that contains link to phylogenetic information within MCMCglmmm models

```
multi_pop_phylo<-multi_pop[multi_pop$species_tree%in%tree$tip.label,]
multi_pop_phylo$animal<-multi_pop_phylo$species_tree
```

Trim phylogeny to only retain species in the new multi_pop dataframe

```
compadre.incpos<-which(tree$tip.label %in% multi_pop_phylo$animal)
compadre.exclude<-tree$tip.label[-compadre.incpos]
compadre.tree<-drop.tip(tree,compadre.exclude)
```

Remove internal node labels from the phylogeny, leaving only tip labels

```
compadre.tree$node.label<-NULL
```

Create final "invas" dataframe for analysis

- Create plant.was and plant.is columns to store information on population "status"
- Create log-transformed versions of the response variables
- Create Invasive Category explanatory variable

1. Native
2. Introduced, native range
3. Introduced, naturalised range
4. Invasive, native range
5. Invasive, naturalised range

```
invas<-multi_pop_phylo

invas$plant.was<-rep("nonnative",dim(invas)[1])
invas$plant.was[invas$Status=="native"]<-"native"
invas$plant.was<-factor(invas$plant.was)
invas$Status.Else[invas$Status.Else=="not introduced"&invas$Status=="introduced"]<-"introduced"
invas$plant.is<-factor(invas$Status.Else)
levels(invas$plant.is)
invas$plant.is[invas$plant.is=="Introduced"]<-"introduced"
invas$plant.is[invas$plant.is=="Not introduced"]<-"not introduced"
invas$plant.is[invas$plant.is=="NATD"]<-NA
invas$plant.is<-factor(invas$plant.is)
levels(invas$plant.is)
levels(invas$plant.was)
invas<-invas[!is.na(invas$plant.is),]

invas$loglambda<-log(invas$lambda.mean)
invas$loginertiaup<-log(invas$inertiaup.mean)
invas$loginertiadown<-log(invas$inertiadown.mean)
invas$logmaxatt<-log(invas$maxatt.mean)
invas$logmaxamp<-log(invas$maxamp.mean)
invas$logreac<-log(invas$reactivity.mean)

invas$invasive.category<-factor(1+(invas$plant.is%in%c("introduced","Introduced"))+(invas$plant.was=="nonnat
ive")+3*(invas$plant.is=="invasive"))
```

Some slight ammendments to invas dataframe

- Create column containing information on matrix dimensions
- only include "Unmanipulated" populations/matrices
- Remove *Miscanthus giganteus*
- Make Invasive Category a factor

```
invas$n<-numeric(dim(invas)[1])
for(i in 1:dim(invas)[1]){
  A<-as.matrix(Matlab2R(as.character(invas$A[i])))
  invas$n[i]<-dim(A)[1]
}
invas<-invas[invas$matrix_treatment=="Unmanipulated",]
invas$invasive.category<-factor(invas$invasive.category)
```

Create persp dataframe

This dataframe contains only a mean of each response variable per species (rather than having multiple populations per species) and therefore enables the simpler analysis used in the main text

```
persp<-aggregate(loglambda~animal+invasive.category,mean,data=invas)
persp$loginertiaup<-aggregate(loginertiaup~animal+invasive.category,mean,data=invas)$loginertiaup
persp$loginertiadown<-aggregate(loginertiadown~animal+invasive.category,mean,data=invas)$loginertiadown
persp$logmaxatt<-aggregate(logmaxatt~animal+invasive.category,mean,data=invas)$logmaxatt
persp$logreac<-aggregate(logreac~animal+invasive.category,mean,data=invas)$logreac
persp$n<-aggregate(n~animal+invasive.category,mean,data=invas)$n
persp$Species<-persp$animal
```

Save the invas and persp dataframes prior to modelling

```
write.csv(invas, paste0(direc,"mcmc_data_invasives.csv"))
write.csv(persp,paste0(direc,"per_species_data.csv"))
```

2.5 R code to fit models to the data

Some information on the Bayesian models used

- Response variables have been logged to ensure that they are Gaussian
- The animal random effect is the phylogeny
- For models of the invas dataframe Species is additionally included as a random effect as we have multiple populations per species
- Burnin of 10% interations and a thinning interval of <0.1% iterations for Markov chains (ideally you need an effective sample size of at least 1000: to be safe can make thinning <0.05%)
- Proper uninformative priors are used (nu is small but >0). In this example we use parameter expansion (alpha.mu and alpha.V) as some of the chains got stuck at 0. We don't need to specify fixed effect priors as MCMCglmm does a good job of picking default one but if we did they'd be specified as B. G is the random effects - the number of G structures should match the number of random effects. If you use random effects interaction or regression, V should be changed to an identity matrix of the same dimension as the number of parameters to estimate. R is the residuals. Parameter expansion for residual priors is not supported (but I've never seen it needed). It doesn't matter what order you put B, G and R in but G1, G2, G3,… must be in the same order as specified for random effects in the model.
- (*verbose=F can stop the models updating in real time*)

2.5.1 Fit Bayesian hierarchical models to the persp dataset (same analysis as the main paper; models the mean for each species)

Model A1 - persp model for log lambda

- Set priors

```
prior1.loglambda<-list(R = list(V = 1, nu=0.001),
               G = list(G1=list(V = 1, nu=0.001, alpha.mu=0, alpha.V=100)))
```

- Run model

```
m1.loglambda<-MCMCglmm(loglambda ~ invasive.category,
                       random=~animal, family="gaussian",
                       prior=prior1.loglambda, data=persp, pedigree=compadre.tree, nodes="TIPS",
                       thin=1000, nitt=1000000, burnin=100000, verbose=T)
```

- Model summary and plots

```
summary(m1.loglambda)
```

```
##
##  Iterations = 100001:999001
##  Thinning interval  = 1000
##  Sample size  = 900
##
##  DIC: 350.4249
##
##  G-structure:  ~animal
##
##        post.mean  l-95% CI u-95% CI eff.samp
## animal  0.002205 7.149e-09 0.008419     1017
##
##  R-structure:  ~units
##
##       post.mean l-95% CI u-95% CI eff.samp
## units    0.1192   0.1027   0.1336      900
##
##  Location effects: loglambda ~ invasive.category
##
##                     post.mean l-95% CI u-95% CI eff.samp  pMCMC
## (Intercept)           0.03045 -0.05708  0.10364    900.0 0.3444
## invasive.category2    0.09280  0.02041  0.16863    900.0 0.0178 *
## invasive.category3   -0.05389 -0.38940  0.29021   1061.9 0.7311
## invasive.category4    0.08915 -0.02937  0.22851    900.0 0.1644
## invasive.category5    0.40403  0.27982  0.53169    756.8 <0.001 **
## ---
## Signif. codes:  0 '***' 0.001 '**' 0.01 '*' 0.05 '.' 0.1 ' ' 1
```

```
plot(m1.loglambda)
```

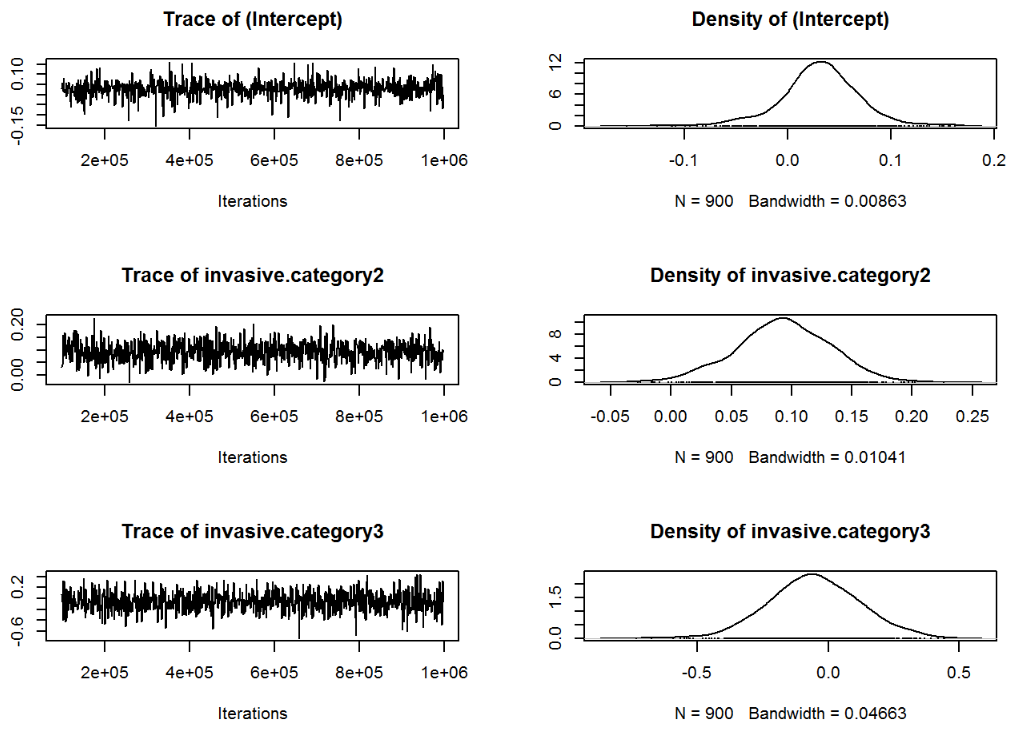

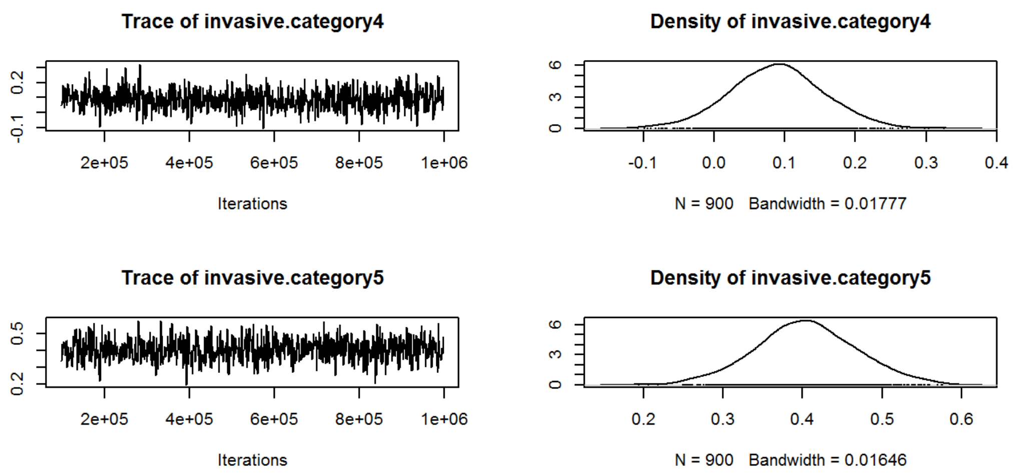

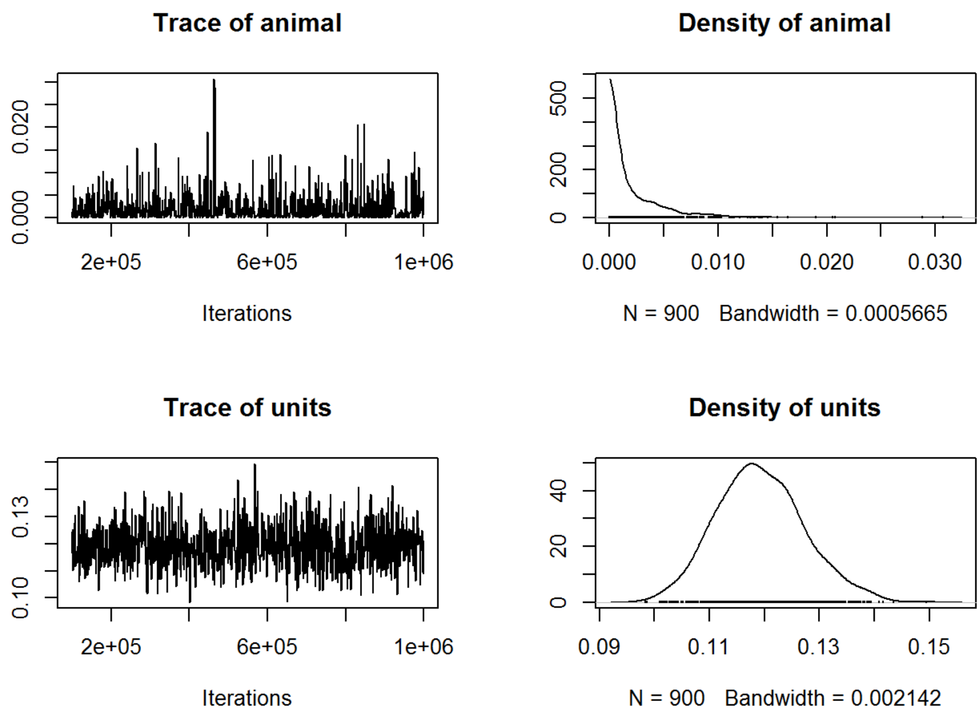

Model A2 - persp model for log inertiaup

- Set priors

```
prior1.inertiaup<-list(R = list(V = 1, nu=0.001),
                G = list(G1=list(V = 1, nu=0.001, alpha.mu=0, alpha.V=100)))
```

- Run model

```
m1.loginertiaup<-MCMCglmm(loginertiaup ~ invasive.category,
                    random=~animal, family="gaussian",
                    prior=prior1.inertiaup, data=persp, pedigree=compadre.tree, nodes="TIPS",
                    thin=1000, nitt=1000000, burnin=10000, verbose=T)
```

- Model summary and plots

```
summary(m1.loginertiaup)
```

```
##
##  Iterations = 10001:999001
##  Thinning interval  = 1000
##  Sample size  = 990
##
##  DIC: 2058.225
##
##  G-structure:  ~animal
##
##        post.mean l-95% CI u-95% CI eff.samp
## animal     1.607  0.07825    3.542      990
##
##  R-structure:  ~units
##
##       post.mean l-95% CI u-95% CI eff.samp
## units     3.891    3.289    4.546      990
##
##  Location effects: loginertiaup ~ invasive.category
##
##                     post.mean l-95% CI u-95% CI eff.samp  pMCMC
## (Intercept)           1.59328  0.22368  2.98812      990 0.0303 *
## invasive.category2  -0.08097 -0.50396  0.41677      990 0.7091
## invasive.category3  -0.50608 -2.34454  1.66941      990 0.6323
## invasive.category4   1.49903  0.70785  2.23146      990 <0.001 **
## invasive.category5   1.80608  0.95215  2.50290      990 <0.001 **
## ---
## Signif. codes:  0 '***' 0.001 '**' 0.01 '*' 0.05 '.' 0.1 ' ' 1
```

```
plot(m1.loginertiaup)
```

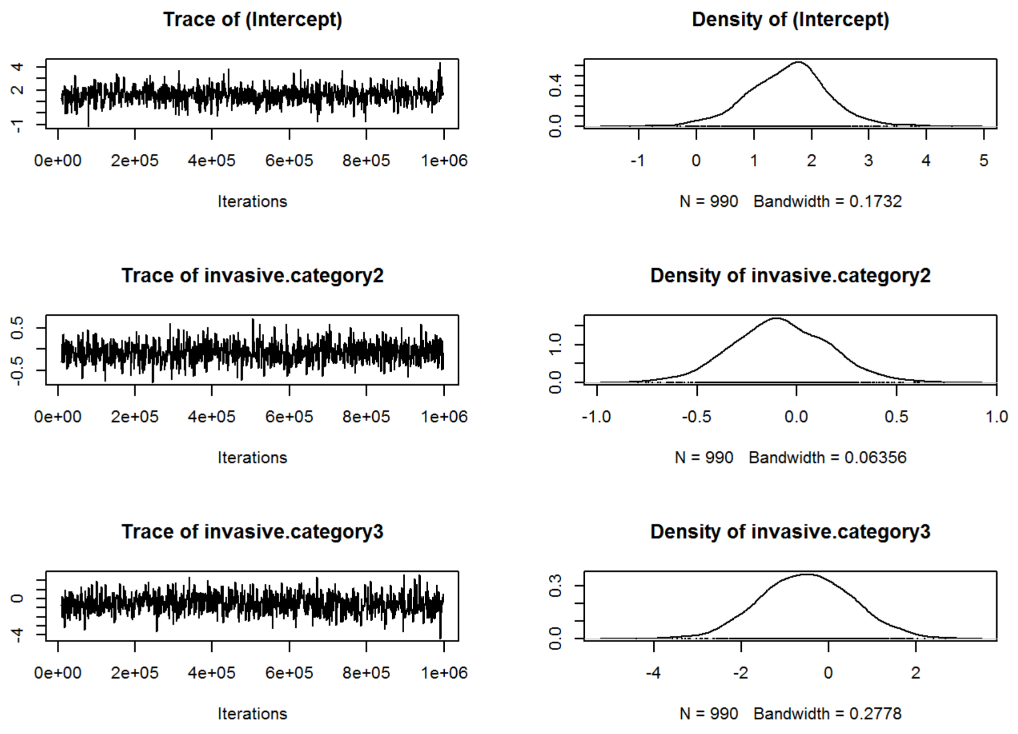

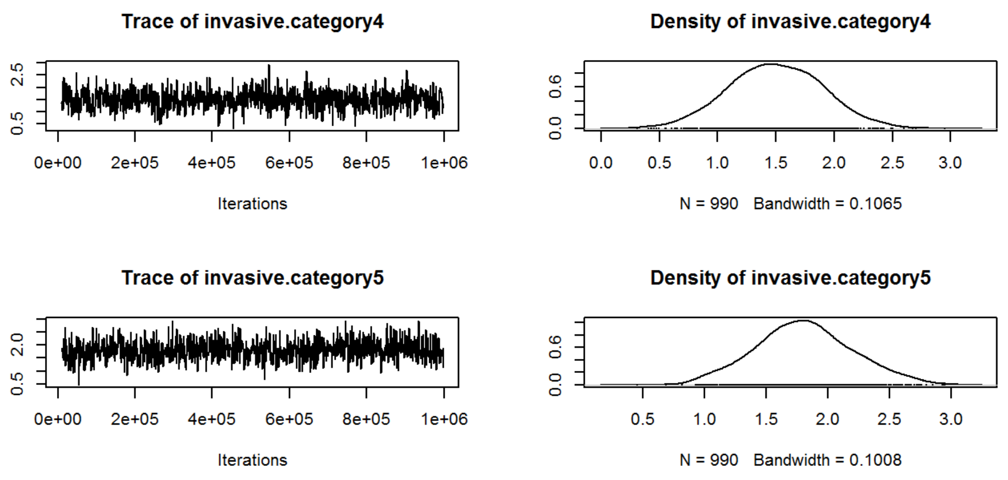

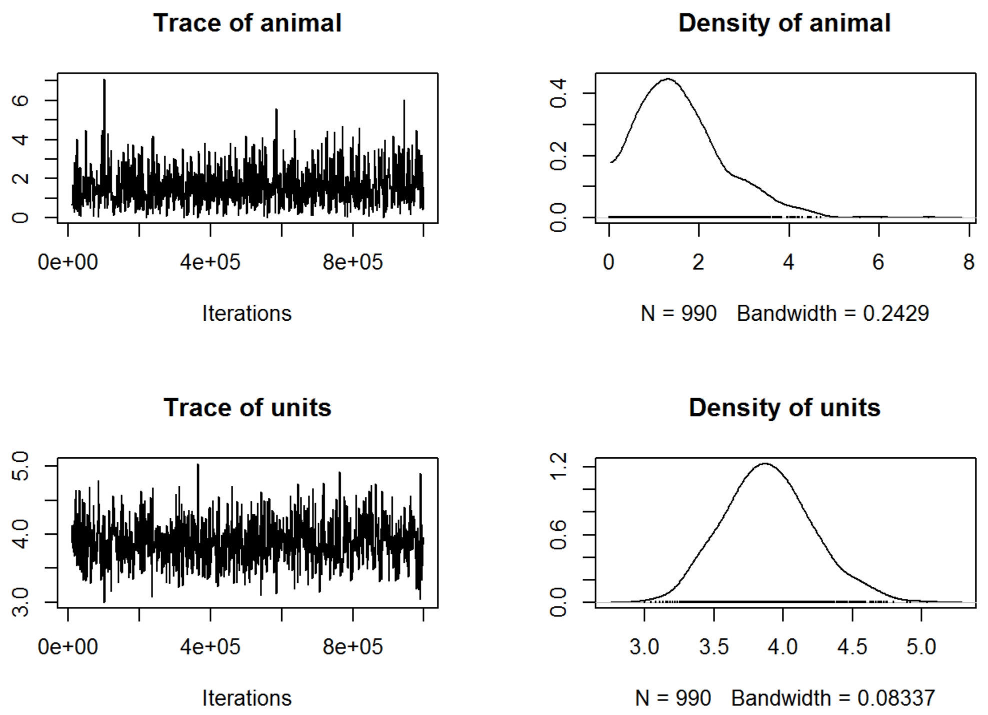

Model A3 - persp model for log inertiadown

- Set priors

```
prior1.inertiadown<-list(R = list(V = 1, nu=0.001),
               G = list(G1=list(V = 1, nu=0.001, alpha.mu=0, alpha.V=100)))
```

- Run model

```
m1.loginertiadown<-MCMCglmm(loginertiadown ~ invasive.category,
                      random=~animal, family="gaussian",
                      prior=prior1.inertiadown, data=persp, pedigree=compadre.tree, nodes="TIPS",
                      thin=1000, nitt=1000000, burnin=10000, verbose=T)
```

- Model summary and plots

```
summary(m1.loginertiadown)
```

```
##
##  Iterations = 10001:999001
##  Thinning interval  = 1000
##  Sample size  = 990
##
##  DIC: 2015.758
##
##  G-structure:  ~animal
##
##        post.mean l-95% CI u-95% CI eff.samp
## animal     2.371   0.2044    5.298     1032
##
##  R-structure:  ~units
##
##       post.mean l-95% CI u-95% CI eff.samp
## units     3.433    2.789    4.056      990
##
##  Location effects: loginertiadown ~ invasive.category
##
##                     post.mean l-95% CI u-95% CI eff.samp   pMCMC
## (Intercept)           -1.7936  -3.3907  -0.2698      990 0.02222 *
## invasive.category2   -0.3886  -0.8162   0.1056     1291 0.11313
## invasive.category3    1.7709  -0.2867   3.6137      990 0.07677 .
## invasive.category4   -0.2336  -0.9769   0.5473      990 0.53535
## invasive.category5   -1.0870  -1.8341  -0.3380      990 0.00202 **
## ---
## Signif. codes:  0 '***' 0.001 '**' 0.01 '*' 0.05 '.' 0.1 ' ' 1
```

```
plot(m1.loginertiadown)
```

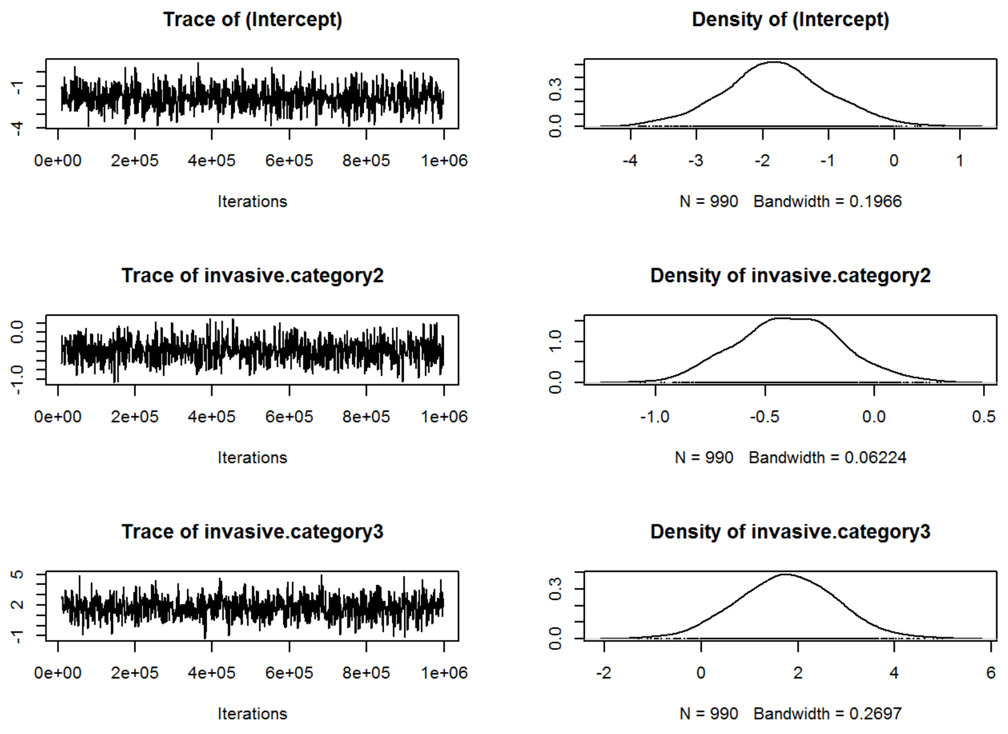

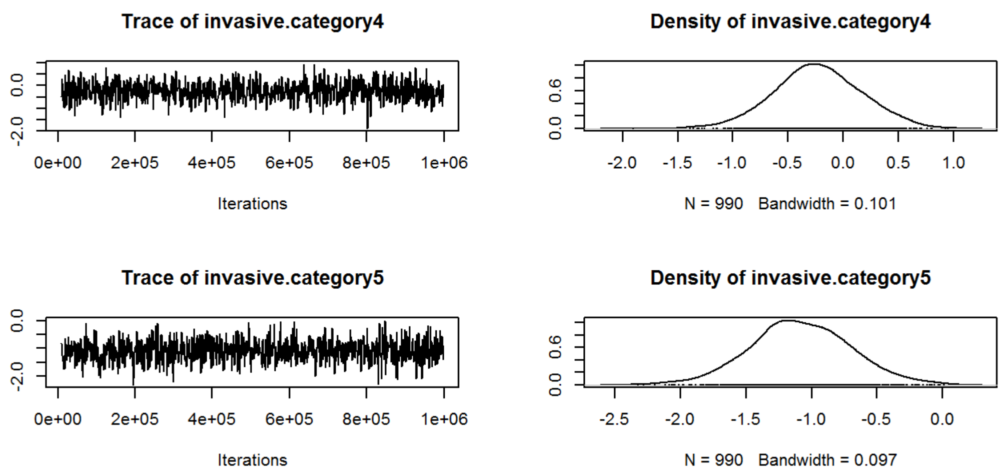

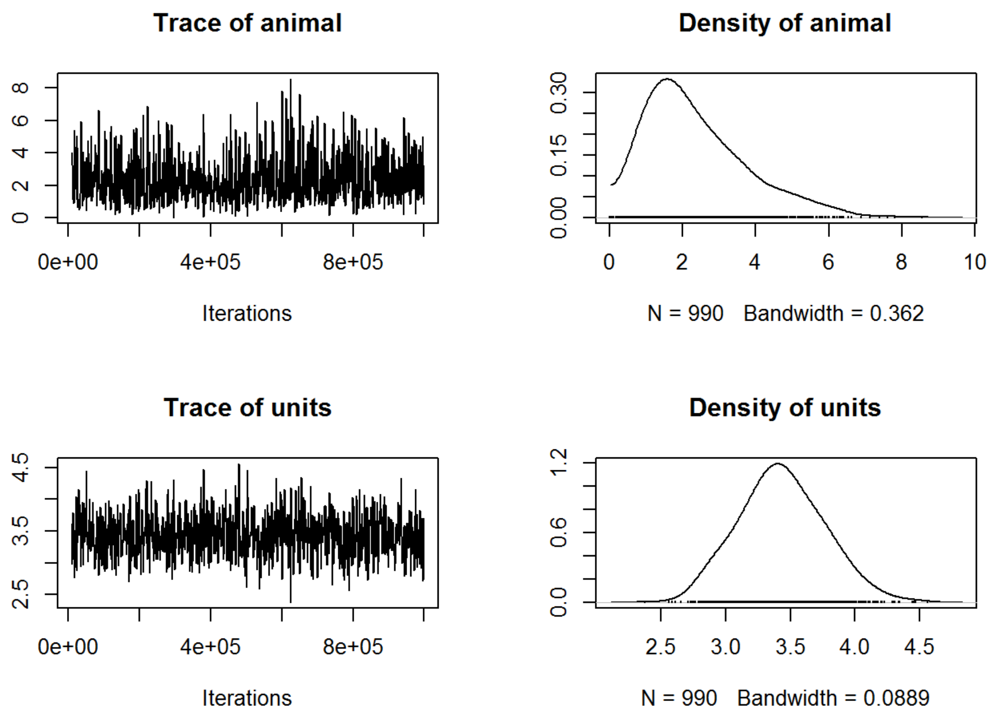

2.5.2 Fit Bayesian hierarchical models to the invas dataset (full analysis including multiple populations of each species)

Model B1 - invas model for log lambda

- Set priors

```
prior2.2<-list(R = list(V = 1, nu=0.001),
               G = list(G1=list(V = 1, nu=0.001, alpha.mu=0, alpha.V=100),
                        G2=list(V = 1, nu=0.001, alpha.mu=0, alpha.V=100)))
```

- Run model

```
m2.loglambda<-MCMCglmm(loglambda ~ invasive.category,
                       random=~Species + animal, family="gaussian",
                       prior=prior2.2, data=invas, pedigree=compadre.tree, nodes="TIPS",
                       thin=1000, nitt=1000000, burnin=10000, verbose=T)
```

- Model summary and plots

```
summary(m2.loglambda)
```

```
##
##  Iterations = 10001:999001
##  Thinning interval  = 1000
##  Sample size  = 990
##
##  DIC: 649.0119
##
##  G-structure:  ~Species
##
##         post.mean l-95% CI u-95% CI eff.samp
## Species    0.0544  0.04051  0.06844     1095
##
##                ~animal
##
##        post.mean  l-95% CI u-95% CI eff.samp
## animal  0.001831 3.498e-08 0.007059    709.2
##
##  R-structure:  ~units
##
##       post.mean l-95% CI u-95% CI eff.samp
## units   0.08261  0.07499  0.09166      990
##
##  Location effects: loglambda ~ invasive.category
##
##                     post.mean  l-95% CI  u-95% CI eff.samp  pMCMC
## (Intercept)          0.030832 -0.037581  0.095302     1104 0.2929
## invasive.category2   0.090549  0.017152  0.158375      990 0.0101 *
## invasive.category3   0.059154 -0.238017  0.419658      990 0.7616
## invasive.category4   0.002237 -0.122940  0.123197      990 0.9778
## invasive.category5   0.379947  0.257661  0.489175      990 <0.001 **
## ---
## Signif. codes:  0 '***' 0.001 '**' 0.01 '*' 0.05 '.' 0.1 ' ' 1
```

```
plot(m2.loglambda)
```

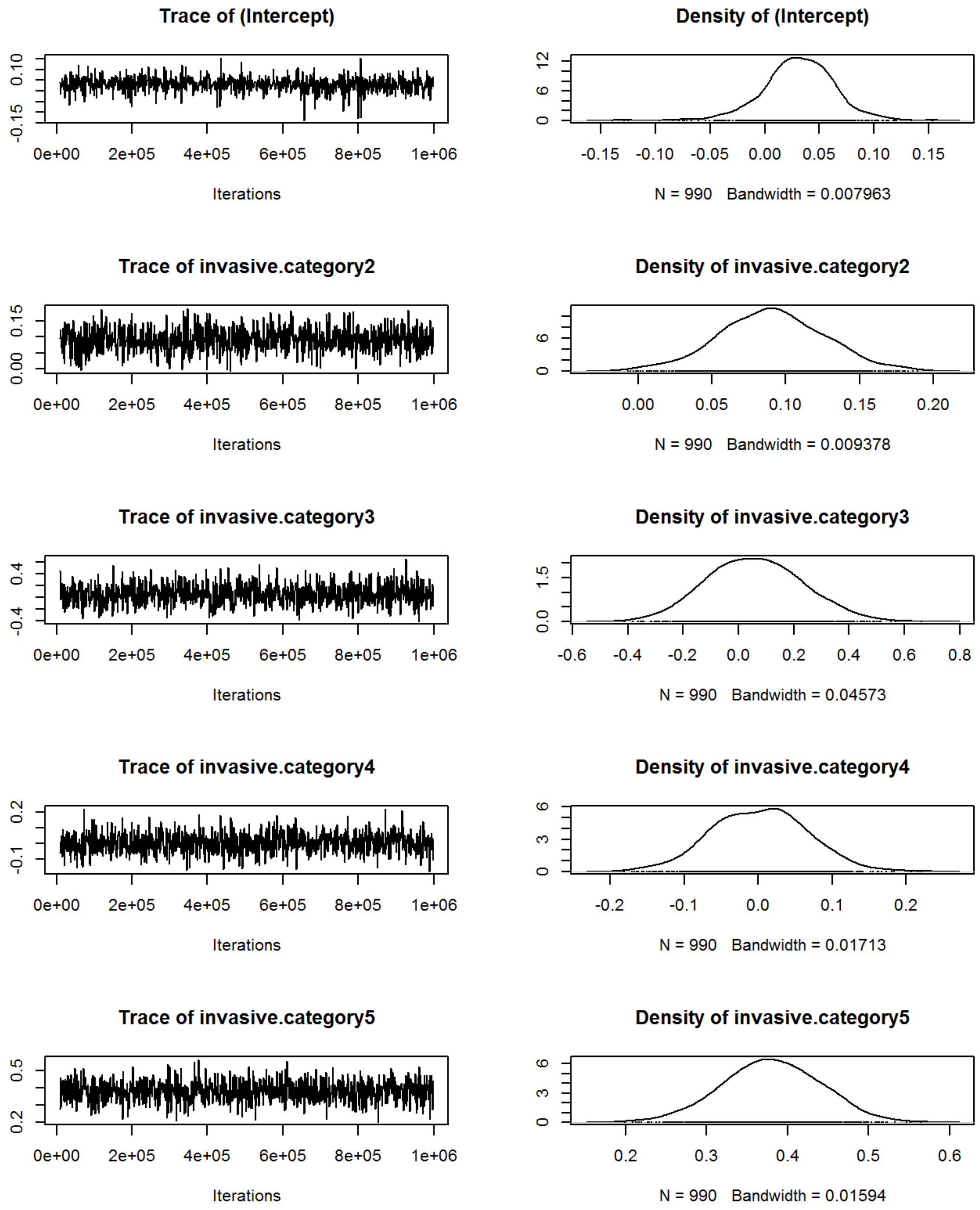

Trace of (Intercept)
Iterations
Density of (Intercept)
N = 990 Bandwidth = 0.007963
Trace of invasive.category2
Iterations
Density of invasive.category2
N = 990 Bandwidth = 0.009378
Trace of invasive.category3
Iterations
Density of invasive.category3
N = 990 Bandwidth = 0.04573
Trace of invasive.category4
Iterations
Density of invasive.category4
N = 990 Bandwidth = 0.01713
Trace of invasive.category5
Iterations
Density of invasive.category5
N = 990 Bandwidth = 0.01594

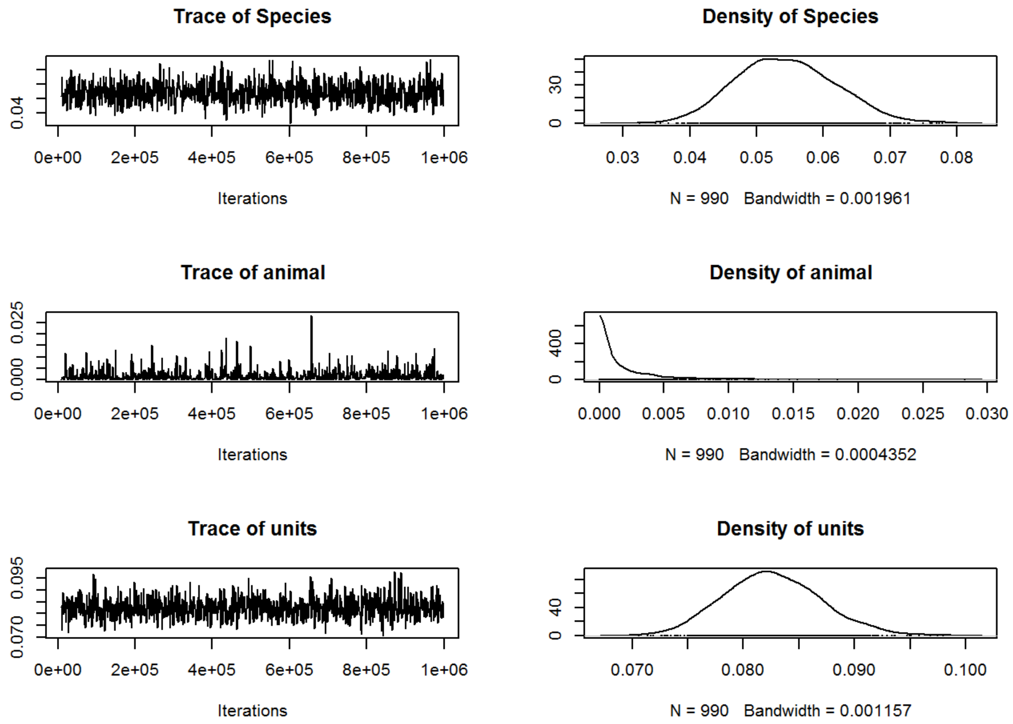

Model B2 - invas model for log inertiaup

- Set priors

```
prior2.inertiaup<-list(R = list(V = 1, nu=0.001),
                 G = list(G1=list(V = 1, nu=0.001, alpha.mu=0, alpha.V=100),
                          G2=list(V = 1, nu=0.001, alpha.mu=0, alpha.V=100)))
```

- Run model

```
m2.inertiaup_full<-MCMCglmm(loginertiaup ~ invasive.category,
                            random=~Species + animal, family="gaussian",
                            prior=prior2.inertiaup, data=invas, pedigree=compadre.tree, nodes="TIPS",
                            thin=1000, nitt=1000000, burnin=10000, verbose=T)
```

- Model summary and plots

```
summary(m2.inertiaup_full)
```

```
##
##  Iterations = 10001:999001
##  Thinning interval  = 1000
##  Sample size  = 990
##
##  DIC: 3078.379
##
##  G-structure:  ~Species
##
##         post.mean l-95% CI u-95% CI eff.samp
## Species     3.417    2.791        4    774.1
##
##                ~animal
##
##        post.mean l-95% CI u-95% CI eff.samp
## animal       1.7 0.003255    3.431    814.4
##
##  R-structure:  ~units
##
##       post.mean l-95% CI u-95% CI eff.samp
## units    0.5869   0.5256   0.6548      990
##
##  Location effects: loginertiaup ~ invasive.category
##
##                     post.mean l-95% CI u-95% CI eff.samp   pMCMC
## (Intercept)           1.56404  0.25930  2.81750      990 0.03030 *
## invasive.category2   -0.05952 -0.50581  0.38636      990 0.76566
## invasive.category3   -0.53962 -2.65919  1.43178      990 0.60808
## invasive.category4    0.82384  0.14707  1.39852      990 0.00808 **
## invasive.category5    2.36880  1.71005  3.04123      990 < 0.001 **
## ---
## Signif. codes:  0 '***' 0.001 '**' 0.01 '*' 0.05 '.' 0.1 ' ' 1
```

```
plot(m2.inertiaup_full)
```

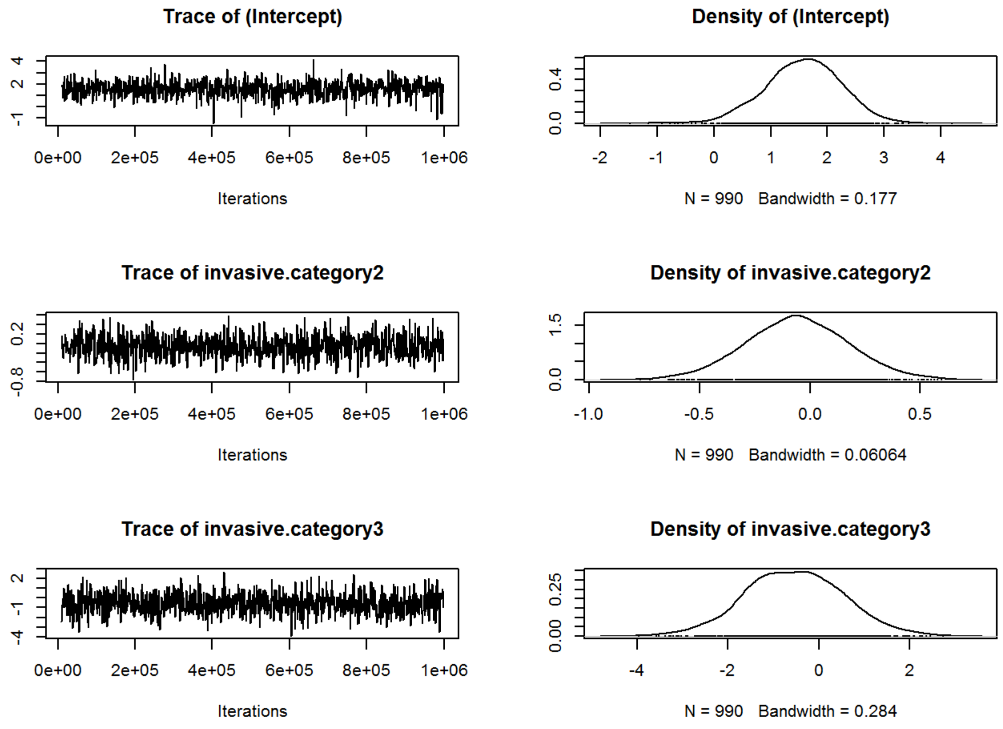

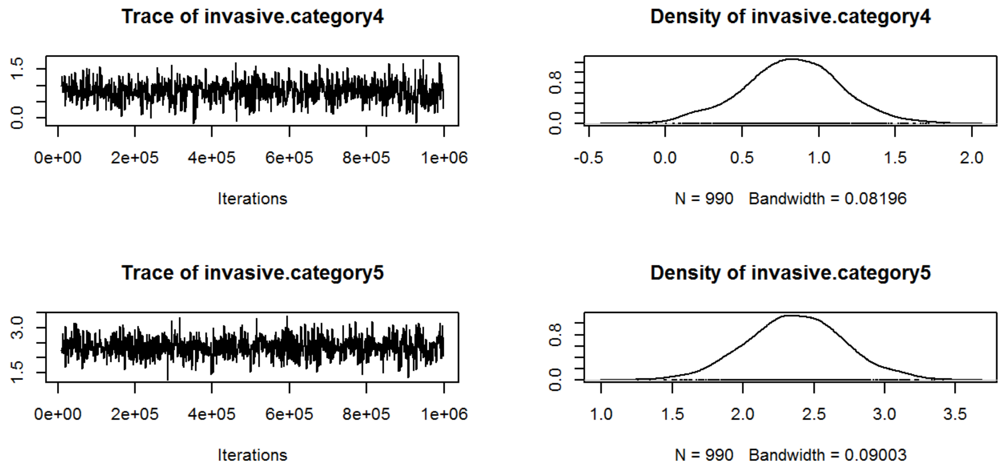

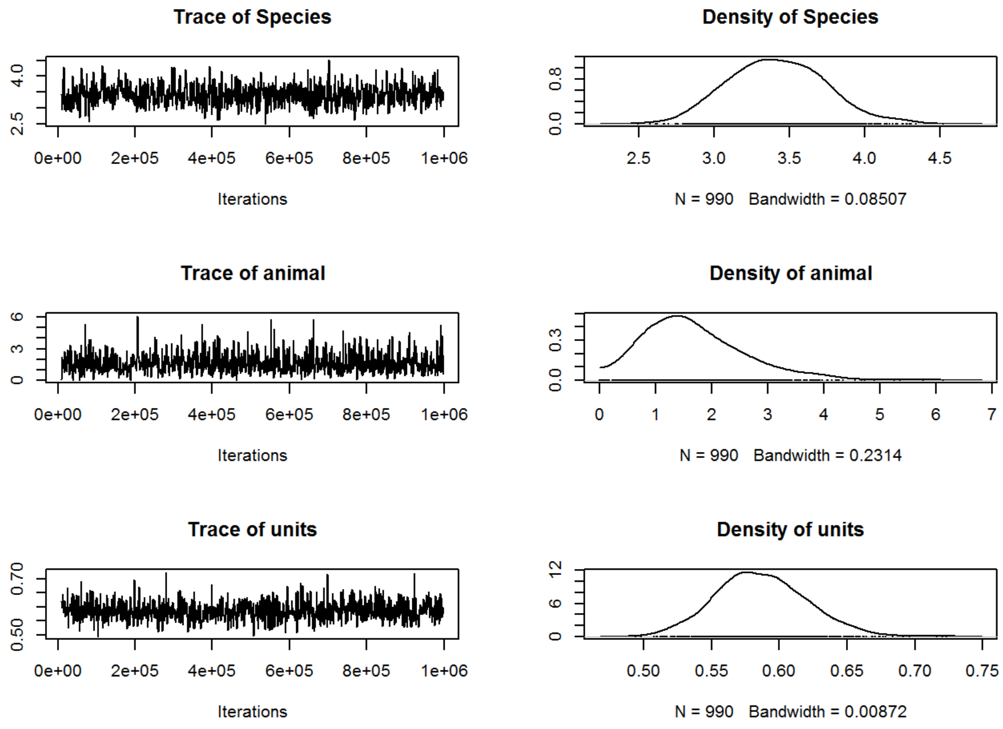

Model B3 - persp model for log inertia down

- Set priors

```
prior2.inertiadown<-list(R = list(V = 1, nu=0.001),
                  G = list(G1=list(V = 1, nu=0.001, alpha.mu=0, alpha.V=100),
                           G2=list(V = 1, nu=0.001, alpha.mu=0, alpha.V=100)))
```

- Run model

```
m2.inertiadown_full<-MCMCglmm(loginertiadown ~ invasive.category,
                              random=~Species + animal, family="gaussian",
                              prior=prior2.inertiadown, data=invas, pedigree=compadre.tree, nodes="TIPS",
                              thin=1000, nitt=1000000, burnin=10000, verbose=T)
```

- Model summary and plots

```
summary(m2.inertiadown_full)
```

```
##
##  Iterations = 10001:999001
##  Thinning interval  = 1000
##  Sample size  = 990
##
##  DIC: 4420.694
##
##  G-structure:  ~Species
##
##         post.mean l-95% CI u-95% CI eff.samp
## Species     2.117    1.626    2.733      990
##
##                ~animal
##
##        post.mean l-95% CI u-95% CI eff.samp
## animal     1.709   0.3399    3.758      990
##
##  R-structure:  ~units
##
##       post.mean l-95% CI u-95% CI eff.samp
## units     2.097    1.875    2.322      990
##
##  Location effects: loginertiadown ~ invasive.category
##
##                    post.mean l-95% CI u-95% CI eff.samp   pMCMC
## (Intercept)          -1.8928  -3.1621  -0.5912    886.2 0.01414 *
## invasive.category2   -0.3366  -0.7254   0.1562    990.0 0.15152
## invasive.category3    1.5187  -0.4606   3.6121    990.0 0.13737
## invasive.category4   -0.3048  -1.0143   0.3798    990.0 0.39394
## invasive.category5   -0.9615  -1.7020  -0.3224   1095.9 0.00606 **
## ---
## Signif. codes:  0 '***' 0.001 '**' 0.01 '*' 0.05 '.' 0.1 ' ' 1
```

```
plot(m2.inertiadown_full)
```

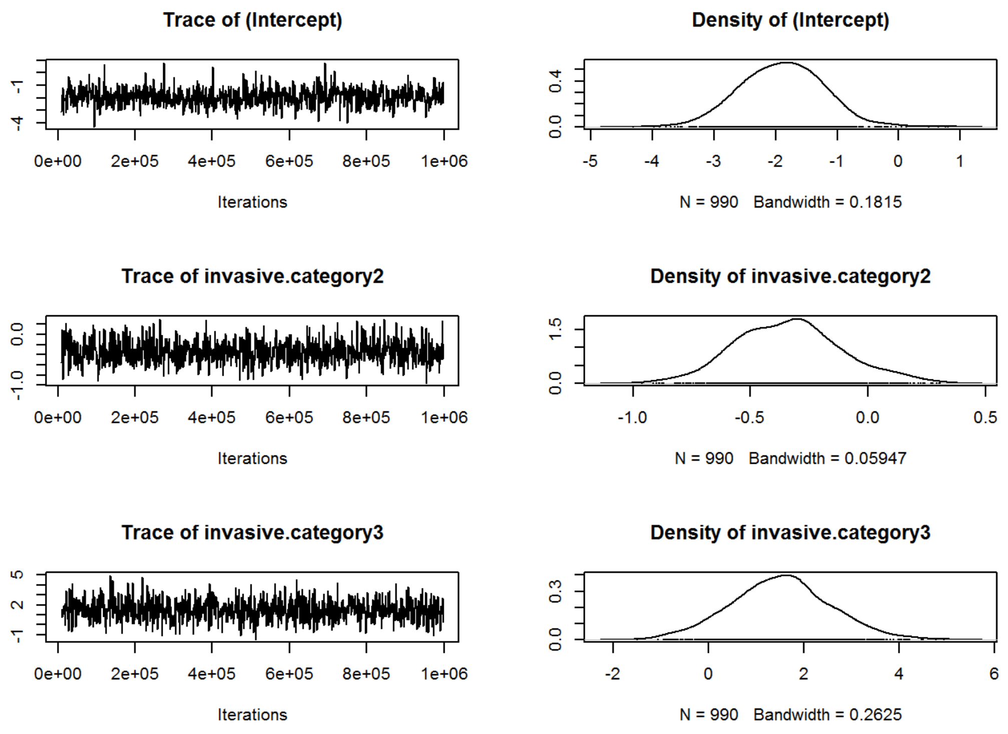

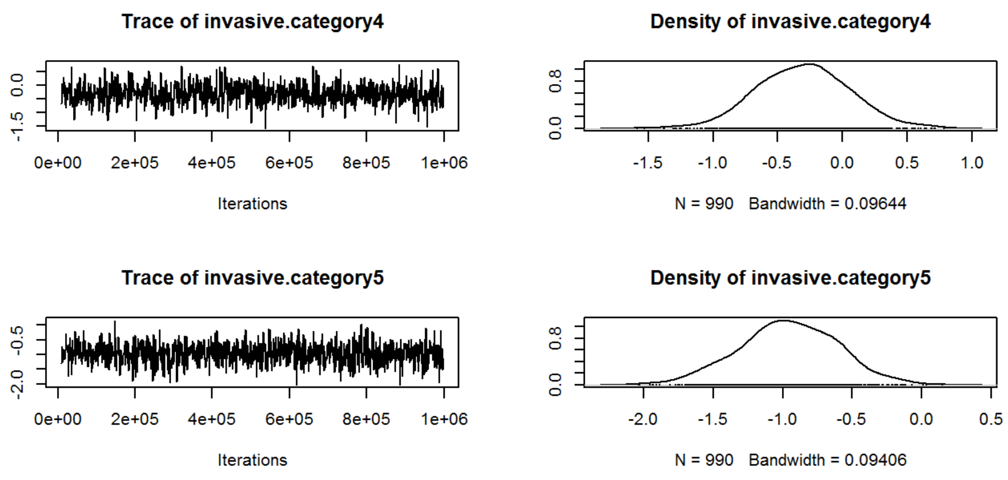

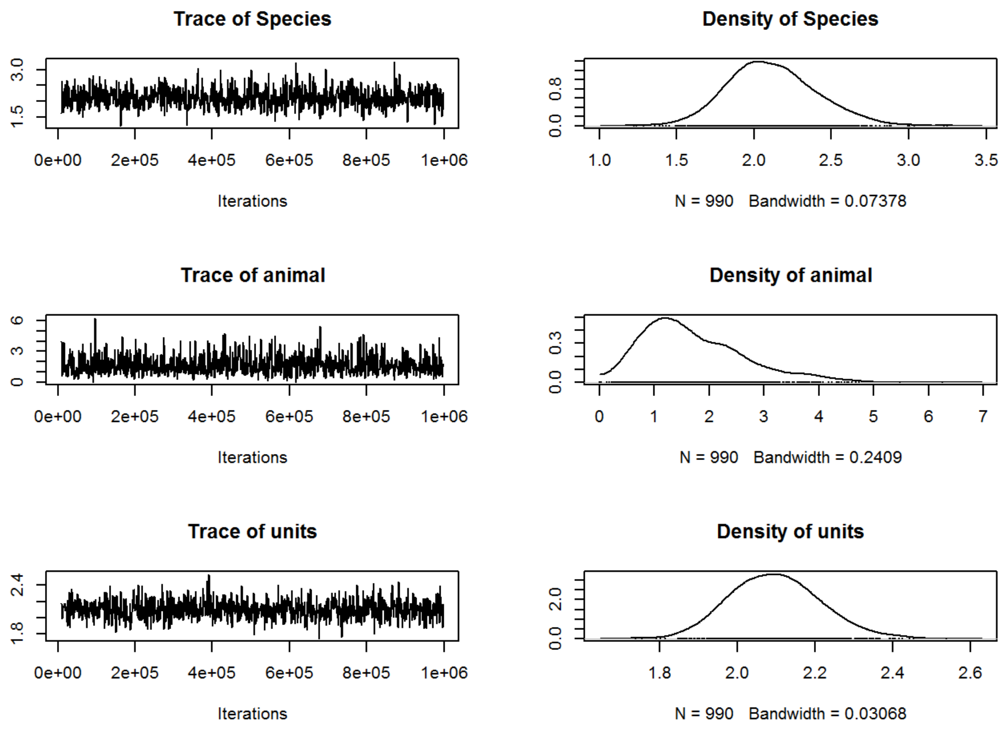

2.5.3 Equivalent non-bayesian models to demonstrate robustness of the results

The first set of models are lme models of the full dataset (invas) with Species as a random effect and no phylogenetic random effect 1.Log lambda 2.Log inertia_up 3.Log inertia_down

```
lmm1<-lme(loglambda~invasive.category-1,random=~1|Species,data=invas)
summary(lmm1)
```

```
## Linear mixed-effects model fit by REML
##  Data: invas
##        AIC      BIC    logLik
##   813.0792 848.3752 -399.5396
##
## Random effects:
##  Formula: ~1 | Species
##         (Intercept)  Residual
## StdDev:   0.2323895 0.2872979
##
## Fixed effects: loglambda ~ invasive.category - 1
##                        Value  Std.Error  DF  t-value p-value
## invasive.category1 0.0360403 0.01850280 669 1.947830  0.0519
## invasive.category2 0.1285332 0.03011434 669 4.268174  0.0000
## invasive.category3 0.0944095 0.17090153 476 0.552420  0.5809
## invasive.category4 0.0403058 0.05872936 669 0.686297  0.4928
## invasive.category5 0.4182648 0.05686848 669 7.354950  0.0000
##  Correlation:
##                    invs.1 invs.2 invs.3 invs.4
## invasive.category2 0.000
## invasive.category3 0.000  0.000
## invasive.category4 0.007  0.009  0.000
## invasive.category5 0.002  0.001  0.000  0.058
##
## Standardized Within-Group Residuals:
##          Min           Q1          Med           Q3          Max
## -11.08751210  -0.22444435  -0.04456832   0.13844004   5.81854671
##
## Number of Observations: 1149
## Number of Groups: 477
```

```
lmm2<-lme(loginertiaup~invasive.category-1,random=~1|Species,data=invas)
summary(lmm2)
```

```
## Linear mixed-effects model fit by REML
##  Data: invas
##        AIC      BIC    logLik
##   3871.383 3906.679 -1928.691
##
## Random effects:
##  Formula: ~1 | Species
##         (Intercept)  Residual
## StdDev:    1.982009 0.7643809
##
## Fixed effects: loginertiaup ~ invasive.category - 1
##                       Value Std.Error  DF   t-value p-value
## invasive.category1 1.951001 0.1175057 669 16.603457  0.0000
## invasive.category2 1.768599 0.1975722 669  8.951655  0.0000
## invasive.category3 1.004502 1.0498235 476  0.956830  0.3391
## invasive.category4 2.657678 0.3027424 669  8.778678  0.0000
## invasive.category5 4.183862 0.3261175 669 12.829310  0.0000
##  Correlation:
##                    invs.1 invs.2 invs.3 invs.4
## invasive.category2 0.003
## invasive.category3 0.000  0.000
## invasive.category4 0.043  0.065  0.000
## invasive.category5 0.022  0.018  0.000  0.283
##
## Standardized Within-Group Residuals:
##         Min          Q1         Med          Q3         Max
## -5.48114264 -0.28371357 -0.06131928  0.28050587  7.65815161
##
## Number of Observations: 1149
## Number of Groups: 477
```

```
lmm3<-lme(loginertiadown~invasive.category-1,random=~1|Species,data=invas)
summary(lmm3)
```

```
## Linear mixed-effects model fit by REML
##  Data: invas
##       AIC      BIC   logLik
##   4695.12 4730.416 -2340.56
##
## Random effects:
##  Formula: ~1 | Species
##         (Intercept) Residual
## StdDev:    1.633369 1.443448
##
## Fixed effects: loginertiadown ~ invasive.category - 1
##                         Value Std.Error  DF    t-value p-value
## invasive.category1 -2.0034366 0.1142581 669 -17.534302   0.000
## invasive.category2 -2.1918753 0.1891075 669 -11.590630   0.000
## invasive.category3 -0.6649263 1.0376249 476  -0.640816   0.522
## invasive.category4 -2.1705751 0.3495795 669  -6.209102   0.000
## invasive.category5 -2.7919901 0.3484106 669  -8.013505   0.000
##  Correlation:
##                    invs.1 invs.2 invs.3 invs.4
## invasive.category2 0.000
## invasive.category3 0.000  0.000
## invasive.category4 0.012  0.017  0.000
## invasive.category5 0.004  0.002  0.000  0.094
##
## Standardized Within-Group Residuals:
##         Min          Q1         Med          Q3         Max
## -10.3784134  -0.1943535   0.1082663   0.3382745   5.6498678
##
## Number of Observations: 1149
## Number of Groups: 477
```

The second set of models are ordinary linear models run in the persp dataset

```
lm1<-glm(loglambda~invasive.category,data=persp)
summary(lm1)
```

```
##
## Call:
## glm(formula = loglambda ~ invasive.category, data = persp)
##
## Deviance Residuals:
##      Min        1Q    Median        3Q       Max
## -1.66551  -0.11297  -0.03449   0.04602   2.64118
##
## Coefficients:
##                    Estimate Std. Error t value Pr(>|t|)
## (Intercept)         0.03633    0.01960   1.853   0.0645 .
## invasive.category2  0.09588    0.03883   2.469   0.0139 *
## invasive.category3 -0.05157    0.17368  -0.297   0.7667
## invasive.category4  0.09268    0.06599   1.404   0.1608
## invasive.category5  0.40450    0.06501   6.222 1.08e-09 ***
## ---
## Signif. codes:  0 '***' 0.001 '**' 0.01 '*' 0.05 '.' 0.1 ' ' 1
##
## (Dispersion parameter for gaussian family taken to be 0.1191183)
##
##     Null deviance: 61.687  on 480  degrees of freedom
## Residual deviance: 56.700  on 476  degrees of freedom
## AIC: 348.6
##
## Number of Fisher Scoring iterations: 2
```

```
lm2<-glm(loginertiaup~invasive.category,data=persp)
summary(lm2)
```

```
##
## Call:
## glm(formula = loginertiaup ~ invasive.category, data = persp)
##
## Deviance Residuals:
##     Min       1Q   Median       3Q      Max
## -3.4797  -1.4410  -0.6178   0.8192  10.4873
##
## Coefficients:
##                    Estimate Std. Error t value Pr(>|t|)
## (Intercept)          1.9571     0.1184  16.523  < 2e-16 ***
## invasive.category2  -0.2173     0.2346  -0.926 0.354961
## invasive.category3  -0.9547     1.0494  -0.910 0.363437
## invasive.category4   1.4174     0.3988   3.555 0.000416 ***
## invasive.category5   1.6248     0.3928   4.136 4.18e-05 ***
## ---
## Signif. codes:  0 '***' 0.001 '**' 0.01 '*' 0.05 '.' 0.1 ' ' 1
##
## (Dispersion parameter for gaussian family taken to be 4.349202)
##
##     Null deviance: 2211.9  on 480  degrees of freedom
## Residual deviance: 2070.2  on 476  degrees of freedom
## AIC: 2079.1
##
## Number of Fisher Scoring iterations: 2
```

```
lm3<-glm(loginertiadown~invasive.category-1,data=persp)
summary(lm3)
```

```
##
## Call:
## glm(formula = loginertiadown ~ invasive.category - 1, data = persp)
##
## Deviance Residuals:
##      Min        1Q    Median        3Q       Max
## -14.8907   -0.5869    0.5199    1.2284    2.4192
##
## Coefficients:
##                    Estimate Std. Error t value Pr(>|t|)
## invasive.category1  -1.9868     0.1148 -17.309  < 2e-16 ***
## invasive.category2  -2.2131     0.1963 -11.275  < 2e-16 ***
## invasive.category3  -0.5812     1.0105  -0.575    0.565
## invasive.category4  -2.0953     0.3690  -5.679 2.37e-08 ***
## invasive.category5  -2.8768     0.3630  -7.926 1.63e-14 ***
## ---
## Signif. codes:  0 '***' 0.001 '**' 0.01 '*' 0.05 '.' 0.1 ' ' 1
##
## (Dispersion parameter for gaussian family taken to be 4.08426)
##
##     Null deviance: 4076.6  on 481  degrees of freedom
## Residual deviance: 1944.1  on 476  degrees of freedom
## AIC: 2048.8
##
## Number of Fisher Scoring iterations: 2
```

3. Guide to supplementary data files

The two files required to replicated the analyses in the paper are provided in the supplementary information. They are:

- COMPADRE filtered Jelbert et al.csv
- phylogeny_test.tre
